# Synergistic action of 6-gingerol as an adjuvant to colistin for susceptibility enhancement in multidrug-resistant *Klebsiella pneumoniae* isolates

**DOI:** 10.1039/d3ra07835c

**Published:** 2024-03-05

**Authors:** Maheswata Sahoo, Dibyajyoti Uttameswar Behera, Rajesh Kumar Sahoo, Saubhagini Sahoo, Suchanda Dey, Enketeswara Subudhi

**Affiliations:** a Centre for Biotechnology, Siksha ‘O’ Anusandhan (Deemed to be University) Kalinga Nagar, Ghatikia Bhubaneswar 751003 Odisha India enketeswarasubudhi@soa.ac.in maheswatasahoo89@gmail.com dibya01bioinfo@gmail.com rajeshkumarsahoo@soa.ac.in saubhagini2015@gmail.com suchandadey1993@gmail.com +91-9861075829

## Abstract

The growing threat to human health posed by multidrug-resistant *Klebsiella pneumoniae* (MDR-KP) indicates an urgent need to develop alternative therapeutic options. The emergence of colistin resistance further adds to the complexity. The study aims to explore *in silico*-screened phytomolecule 6-gingerol, the most potent active constituent of ginger, as an adjuvant to restore sensitivity in MDR-KP isolates to colistin. The screening of phytocompounds of *Zingiber officinale* were obtained from the spiceRx database, and molecular docking with efflux pump protein AcrB was performed using Schrödinger's Glide program. The synergistic and bactericidal effects of 6-gingerol in combination with colistin against MDR-KP isolates were determined following broth micro-dilution (MIC), checkerboard assay, and time-kill study. 6-Gingerol showed a good binding affinity with AcrB protein (−9.32 kcal mol^−1^) and followed the Lipinski rule of (RO5), demonstrating favourable drug-like properties. Further, the synergistic interaction of 6-gingerol with colistin observed from checkerboard assays against efflux-mediated colistin resistance MDR-KP isolates reveals it to be a prospectus adjuvant. The time-killing assays showed the effect of 6-gingerol in combination with colistin to be bactericidal against MSK9 and bacteriostatic against MSK4 and MSK7. Overall, the study provides insights into the potential use of 6-gingerol as a safe and easily available natural product to treat multidrug-resistant *K. pneumoniae* infections combined with colistin but needs *in vivo* toxicity evaluation before further recommendations can be made.

## Abstract 2

The rapid emergence of MDR *Klebsiella pneumoniae* demands alternative therapy. 6-Gingerol is a natural phytocompound that has synergistic and bactericidal effects against MDR-KP isolates. It exhibited promising EPI activity to stop colistin efflux.

## Graphical abstract

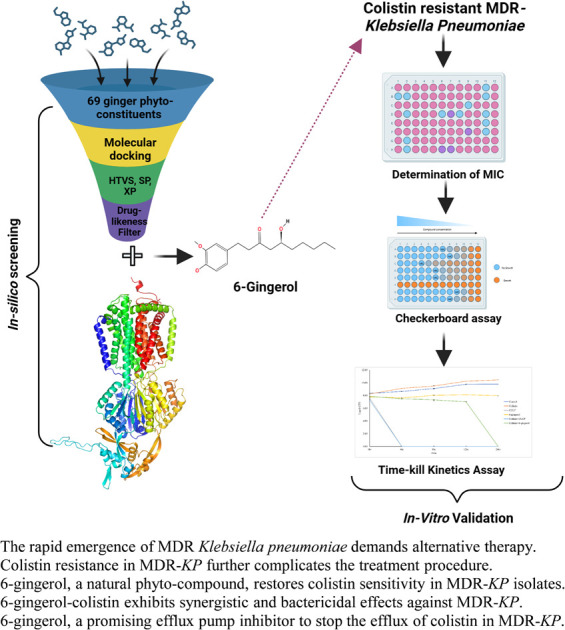

## 1. Introduction

The global emergence of multidrug-resistant (MDR) Gram-negative bacteria poses a growing threat to human health. According to the World Health Organization (WHO), 700 000 annual deaths are associated with MDR bacteria, which could reach up to 10 million by 2050.^1–3^ WHO has earmarked some highly resistant Critical Priority pathogens under the acronym ESKAPE, which include *Enterococcus faecium*, *Staphylococcus aureus*, *Klebsiella pneumoniae*, *Acinetobacter baumannii*, *Pseudomonas aeruginosa*, and *Enterobacter species*.^4^ Among them, *Klebsiella pneumoniae* is the most frequently reported healthcare-associated MDR pathogen that causes urinary tract infections, pneumonia, meningitis, and sepsis.^5^ The frequent use of polymyxin E (colistin) in recent decades as a last resort drug, driven by its efficacy in achieving satisfactory serum levels and low minimum inhibitory concentrations (MIC) against carbapenem-resistant *K. pneumoniae*, has led to the emergence of resistance.^4,6^ The over-expression of the efflux pump systems helps extrude antibacterial molecules out of the bacterial cell, thereby reducing their concentrations to an insufficient quantity for proven effectiveness. It is becoming the predominant mechanism behind the emergence of MDR.^4,7^ Bacterial efflux pumps are essential for drug extrusion and play a role in their virulence and adaptive responses, according to recent clinical and laboratory data.^8^ Phenotypic profiling has shown that exposure to antimicrobial drugs frequently causes complex bacterial reactions, including altered expressions of several genes encoding the transporters.^9^ Bacterial efflux pumps are recognized as either primary active transporters that use ATPs as an energy source or secondary active transporters that are obtained as a result of the electrochemical potential difference produced by pumping out Na^+^ and H^+^ outside the membrane.^10^ Hence, it is necessary to select specific inhibitors of efflux pumps, which can be combined with conventional antibiotics to restore their use.

The absence of novel antibiotics has led to exploring alternative sources, most preferably the secondary metabolites with therapeutic properties, such as terpenoids, phenolics, and alkaloids from medicinal plants, due to their historical use, low cost, and easy availability.^11^ Recently, these plants have gained considerable attention as approximately 40% of current medications are derived from phytochemicals.^12^ Phytochemicals are known to exert a direct antimicrobial effect and improve the efficacy of conventional antibiotics when used in combinations.^11,12^ Furthermore, these plant-derived compounds can interact with critical stages of the pathogenic process, reducing the potential of bacteria to develop resistance.^12^ Consequently, combining these compounds with conventional antibiotics seems promising by allowing their reutilization, which has lost effectiveness due to the overactivity of the efflux pump system in Gram-negative bacteria.^13,14^ Therefore, restoring the sensitivity of bacteria to colistin using potential phytochemicals as an adjuvant could considerably improve therapeutic outcomes and may be a potential approach for treating infections caused by colistin-resistant MDR-KP.^12^

Previous research has demonstrated that *Zingiber officinale* phytoconstituents are promising candidates for treating bacterial infections and biofilm inhibition; these have also been used safely in home remedies for a long time.^15,16^*In silico* screening has been a proven method to narrow down a larger library of molecules from the Spice Rx database to a single molecule against the target. In this study, we selected 6-gingerol based on molecular docking and its pharmacological properties for additional *in vitro* investigation. 6-Gingerol is one of the most abundant natural polyphenols found in ginger rhizomes that exhibits multiple biological activities, including anti-inflammatory, antitumor, antioxidant, and antibacterial. Studies confirmed that the 6-gingerol has anti-biofilm activities against drug-resistant *Candida albicans*^15,17^ and quorum-sensing inhibition activity in *P. aeruginosa*.^18^ Furthermore, 6-gingerol is highly effective in overcoming the complications of multidrug resistance associated with chemotherapeutic agents.^19^

Based on the above facts, in this study, we collected 23 MDR-KP from a six-month surveillance study of the Central Laboratory of IMS and SUM hospital, Bhubaneswar, Odisha. These were subjected to a series of *in vitro* analyses: the broth micro-dilution technique, checkerboard assay, and time-kill kinetics to determine its role as an adjuvant to colistin for enhancing susceptibility in MDR-KP. This study provides valuable insights into using the easily available natural molecule 6-gingerol to inhibit the growth of MDR-KP bacteria and enhance the efficacy of routine antibiotics. These findings represent a significant contribution to the emerging research on the validation of the use of medicinal plant constituents for treating MDR infections as an efflux pump inhibitor.

## 2. Material and methods

### 2.1. Chemicals and solvents

Cation adjusted Mueller Hinton broth (CaMHB), Luria Bertani agar (LBA), Luria Bertani broth (LB), Tryptic soy agar (TSA), dimethyl sulfoxide (DMSO), carbonyl cyanide *m*-chloro phenylhydrazine (CCCP), ethidium bromide (EtBr), colistin sulfate, and triphenyl tetrazolium chloride (TTC) were procured from Himedia, India, and 6-gingerol was purchased from Sigma-Aldrich, India.

### 2.2. *In silico* assay

#### 2.2.1. Molecular docking

The *K. pneumoniae* AcrB protein, part of efflux transporters, was selected for molecular docking. Owing to the unavailability of its 3D structure in the Protein Data Bank (PDB), homology modeling was performed using the RoseTTAFold server (https://robetta.bakerlab.org). The modeled AcrB protein underwent structural refinement using the Protein Preparation Wizard. The ligand preparation involved obtaining a library of 69 Phytocompounds of *Z. officinale* from the spice Rx database (https://cosylab.iiitd.edu.in/spicerx) and processing their 3D structures using the LigPrep module.^20^ The Schrödinger's Glide program performed molecular docking between the selected ligands and AcrB.^21^ The docking analysis employed three steps of docking modes in Glide: HTVS, SP, and XP, with XP GScore used for ranking.^22^ To identify potential phytocompounds, these were subjected to drug-likeness filters in the SwissADME database (http://www.swissadme.ch).

### 2.3. *In vitro* assay

#### 2.3.1. Sample collection and determination of efflux pump activity

During a surveillance study from July 2021 to January 2022, we collected twenty-three colistin-resistant MDR *Klebsiella pneumoniae* from our University Hospital, Institute of Medical Sciences and SUM Hospital, Bhubaneswar, Odisha, India. Further, to screen out efflux-mediated colistin resistance MDR-KP, the EtBr cartwheel test was performed as described by Behera *et al.* (2023).^23^ The isolates (10^6^ CFU mL^−1^) were streaked on TSA plates supplemented with EtBr (0–2.5 mg L^−1^) following the cartwheel pattern. *K*. *pneumoniae* SDL79 (ref. 24) and *E. coli* ATCC 25922 were used as positive and negative controls, respectively. All isolates were examined under UV light, and the result was interpreted from the minimum concentration of EtBr that yielded fluorescence.

#### 2.3.2. Antibiotic susceptibility study and bacterial identification

The susceptibility of the selected isolates was determined using the VITEK2 method against a range of antibiotics, excluding colistin. Furthermore, the colistin susceptibility was determined by minimum inhibitory concentrations (MIC) using the broth microdilution method as previously described.^16^ The concentrations of colistin ranging from 1024 to 1 μg mL^−1^ were used for the MIC study. The experiments were conducted in triplicates, and results were interpreted as per CLSI (2020) breakpoints against Enterobacteriaceae. The *E. coli* ATCC 25922 was used as a control strain for the antimicrobial susceptibility study. Furthermore, the species identification in the selected isolates was performed using the 16S rRNA gene sequencing method through PCR amplification.^25^

#### 2.3.3. Inhibition of efflux pump activity by CCCP

CCCP was then used as an efflux pump inhibitor to confirm efflux pump activity in colistin resistance isolates.^26^ The MIC of CCCP was evaluated by broth microdilution method ranging from 128 to 0.5 μg mL^−1^. The 1/2MIC of CCCP was used to investigate the presence of an efflux pump. The colistin sensitivity assay was performed in the CaMHB in the presence and absence of CCCP. The enhancement of sensitivity to colistin in the presence of CCCP indicates that the efflux pump mediated the colistin resistance in the isolates.

#### 2.3.4. Determination of MIC

The antimicrobial activity of 6-gingerol was evaluated by the broth microdilution method using a micro-titer plate as previously described.^16^ The experiments were performed in triplicate to minimize experimental error.

#### 2.3.5. Checkerboard assay

The interaction of 6-gingerol with colistin against MDR-KP was determined using the checkerboard method as previously described.^23^ The effectiveness of the antimicrobial combinations was determined using the fractional inhibitory concentration index (FICI). The FIC index was calculated using the formula:
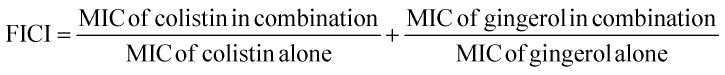


If the FICI value ≤0.5, ≤1, ≤4, and >4, there would be synergistic, additive, indifferent, and antagonistic interactions between 6-gingerol and colistin.

#### 2.3.6. Time-kill assay

The *in vitro* bactericidal activity of 6-gingerol (1/4MIC) in combination with colistin against MDR-KP was studied as previously described.^23^ The early-log phase of the bacterial culture was inoculated in combination with colistin+6-gingerol. At various time intervals (0 h, 4 h, 8 h, 12 h, and 24 h), the culture was retrieved, plated on LB agar, and incubated at 37 °C for 24 h. The time-kill curves were obtained by plotting the log_10_ colony forming unit per mL (CFU mL^−1^) against time (h).

## 3. Results and discussion

### 3.1. *In silico* analysis

**Fig. 1:**
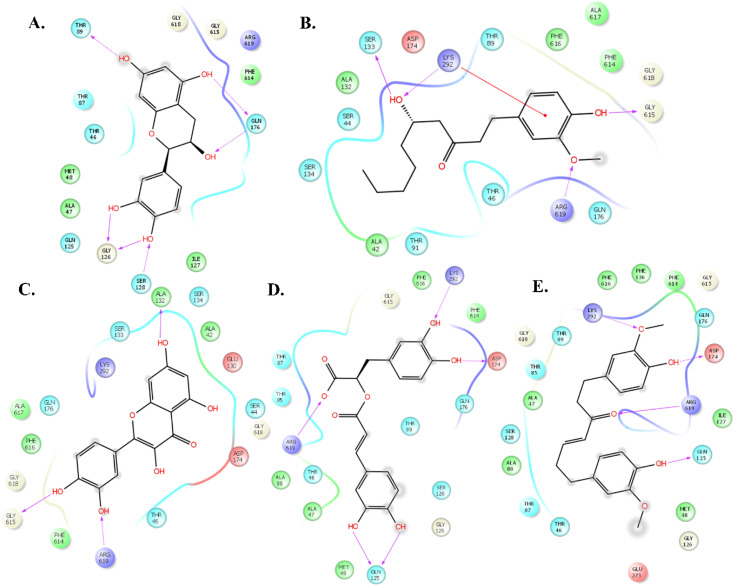
The top-ranked compounds from the XP method bind to the active site of AcrB. The binding site ligand interaction is represented as stick models and coloured by the elements. PyMOL was used to prepare the figures. The figure illustrates the two-dimensional interaction of (A) epicatechin, (b) 6-gingerol, (c) quercetin, (d) rosmarinic acid, and (e) gingerenone A with the key amino acid residues.

The molecular docking study of 69 phyto compounds with AcrB protein screened out the top five compounds with high XP docking scores. Epicatechin exhibited a binding affinity of −10.78 kcal mol^−1^, forming four hydrogen bonds with SER128, GLY126, ASP 276, and ARG 619.6-Gingerol displays a binding affinity of −9.32 kcal mol^−1^, establishing four hydrogen bonds with ASP174, GLN176, LYS292, and ARG619. Quercetin demonstrates a binding affinity of −9.12 kcal mol^−1^, forming five hydrogen bonds with ASP83, THR87, THR91, SER133, and LYS292. Rosmarinic acid exhibited a binding affinity of −8.52 kcal mol^−1^, establishing four hydrogen bonds with THR89, GLN125, PHE616, and ARG619. Gingerenone A has a binding affinity of −8.45 kcal mol^−1^, forming three hydrogen bonds with GLN125, LYS292, and ARG619 (Fig. 1). Additionally, based upon drug-likeness filters, such as Log S (ESOL), molecular weight, number of heavy atoms, number of rotatable bonds, Lipinski rule of (RO5), GI absorption, BBB permeant, and bioavailability score of the five compounds, 6-gingerol demonstrated favourable drug-like properties (Table 1). Thus, 6-gingerol was further considered for the *in vitro* antimicrobial efficacy study.

**Table 1:** Molecular Docking scores of top five phyto-compounds of ginger plant with AcrB efflux pump protein and their ADMET properties^a^

Compound name	Docking score	ADMET screening properties							
Phytocompounds	XP score (kcal mol^−1^)	Log *S* (ESOL)	Molecular weight	Number of heavy atoms	Number of rotatable bonds	Lipinski (RO5)	GI absorption	BBB permeant	Bioavailability score
Epicatechin	−10.78	Soluble	290.27	21	1	0	High	No	0.55
6-Gingerol	−9.32	Soluble	294.39	21	10	0	High	Yes	0.55
Quercetin	−9.12	Soluble	302.24	22	1	0	High	No	0.55
Rosmarinic acid	−8.52	Soluble	360.31	26	7	0	Low	No	0.56
Gingerenone A	−8.45	Moderately soluble	356.41	26	9	0	High	Yes	0.55

a RO5: Rule of five; XP: extra precision; ESOL: estimated solubility; ADMET: absorption, distribution, metabolism, excretion, and toxicity; GI absorption: gastrointestinal absorption; BBB permeant: blood–brain barrier permeant.

### 3.2. *In vitro* analysis

#### 3.2.1. Isolation and identification of efflux pump-mediated resistant MDR-KP

**Fig. 2:**
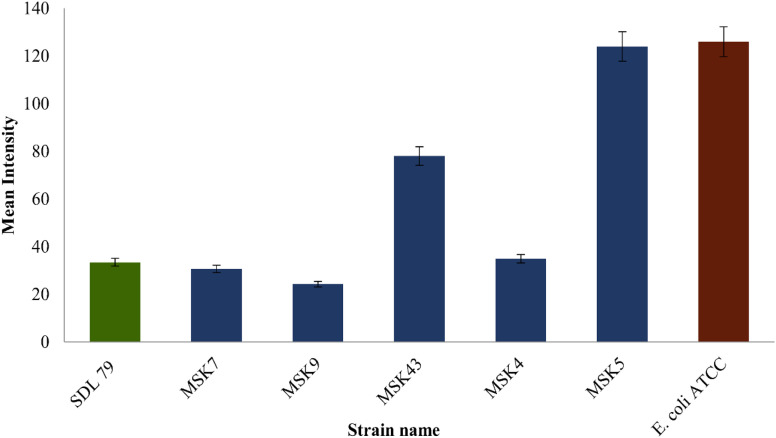
Accumulation and efflux activity on EtBr agar plates containing MSK7, MSK9, MSK43, MSK4, and MSK5 multidrug resistance *Klebsiella pneumoniae* strains; here SDL 79 was taken as positive control and *E. coli* ATCC 25922 was the negative control.

Twenty-three colistin-resistant MDR-KP were collected from our university hospital during the surveillance study. These isolates were tested for the efflux pump activity using the cartwheel method. The fluorescence emitted by TSA plates containing 2.5 μg mL^−1^ EtBr was inversely proportional to their capacity to expel EtBr. Three isolates (MSK4, MSK9, and MSK7) had lower fluorescence intensity, indicating high efflux activity compared to the control strains (Fig. 2). The clinical isolates MSK4, MSK9, and MSK7 were then identified as *K. pneumoniae* through 16S rRNA gene amplification and sequencing.^27^ The 16S rRNA sequences were then submitted to the GenBank database, and the accession numbers OR056343, OR056344, and OR056345 were obtained. The antibiotic sensitivity study conducted by VITEK2 revealed that these isolates were resistant to β-lactam, β-lactam+β-lactamase inhibitor, cephalosporin, carbapenem, aminoglycosides, fluoroquinolone and nitrofuran groups of antibiotics (Table 2). These three isolates demonstrated resistance to colistin by the broth microdilution method, and the MIC range was between 256 and >1024 μg mL^−1^. Furthermore, the MICs of CCCP of these isolates ranged from 8 to 16 μg mL^−1^. These isolates showed a 16-fold reduction in colistin MIC values at the 1/2 MIC of CCCP combined with colistin (Table 3).

**Table 2:** Vitek 2 identification of three isolates (MSK4, MSK7 and MSK9) according to the CLSI/EUCAST guideline^a^

Antimicrobial		MSK4 (MIC)	Interpretation	MSK7 (MIC)	Interpretation	MSK9 (MIC)	Interpretation
β-lactam	Ampicillin	≥32	R	≥32	R	≥32	R
β-lactam+ β-lactamase inhibitor	Piperacillin/tazobactam	≥128	R	≥128	R	≥128	R
	Amoxicillin/clavulanic acid	≥32	R	≥32	R	≥32	R
Cephalosporin	Cefepime	≥64	R	32	R	32	R
	Cefoperazone/sulbactam	≥64	R	≥32	*R	≥64	R
	Ceftriaxone	≥64	R	≥64	R	≥64	R
	Cefuroxime	≥64	R	≥64	R	≥64	R
	Cefuroxime axetil	≥64	R	≥64	R	≥64	R
Carbapenem	Meropenem	≥16	R	8	R	8	R
	Imipenem	8	R	≤0.25*	*I	≤0.25*	*I
	Ertapenem	≥8	R	4	R	≥8	R
Aminoglycosides	Amikacin	≥64	R	≥64	R	≥64	R
	Gentamicin	≥16	R	≥16	R	≥16	R
Fluroquinolone	Ciprofloxacin	≥4	R	≥4	R	≥8	R
	Nalidixic acid	≥32	R	≥32	R	≥32	R
Nitrofuran	Nitrofurantoin	128	R	64	I	128	R
Polymyxin	Colistin	≥16	R	≥8	R	≥16	R
Folate pathway	Trimethoprim/sulfamethoxazole	≤20	S	≥320	R	≥320	R
Glycylcyclines	Tigecycline	2	S	≤0.5	S	2	S

a S: susceptible; I: intermediate; R: resistant; MIC: minimum inhibitory concentration.

**Table 3:** Summary of fold change in colistin MIC after adding CCCP and in colistin MIC after adding 6-gingerol, by *in vitro* combinational method with their FICI values, against three multi drug resistance *Klebsiella pneumoniae* strains^a^

Strain name	MIC colistin (μg mL^−1^)	MIC CCCP (μg mL^−1^)	MIC 6-gingerol (μg mL^−1^)	MIC colistin + CCCP (μg mL^−1^)	Fold reduction (colistin)	MIC colistin+6-gingerol (μg mL^−1^)	Fold reduction (colistin)	FICI	Outcome
MSK7	512	16	512	32/2	16	64/32	8	0.18	Synergistic
MSK4	256	16	128	16/4	16	8/16	32	0.15	Synergistic
MSK9	≥1024	16	512	64/2	16	128/64	8	0.25	Synergistic

a FICI: fractional inhibitory concentration index; MIC: minimum inhibitory concentration; CCCP: carbonyl cyanide *m*-chlorophenylhydrazine.

#### 3.2.2. *In vitro* antimicrobial efficacy of 6-gingerol and its combination with colistin

6-Gingerol was tested against the clinical isolates MSK4, MSK9, and MSK7 to support the *in silico* prediction. The MIC value ranged from 128 to 512 g mL^−1^ against all the isolates. It demonstrated that the MIC of 6-gingerol was lower than the MIC of colistin against MSK4, MSK9, and MSK7 isolates. The enhancement in susceptibility of MSK4, MSK9, and MSK7 to colistin in combination with 6-gingerol was then evaluated. Our findings indicate that 6-gingerol synergistically interacted with colistin FICIs ranging from 0.18 to 0.28 (Table 3) since 6-gingerol exhibited up to an 8-to-32-fold reduction in the MIC of colistin against these isolates.

**Fig. 3:**
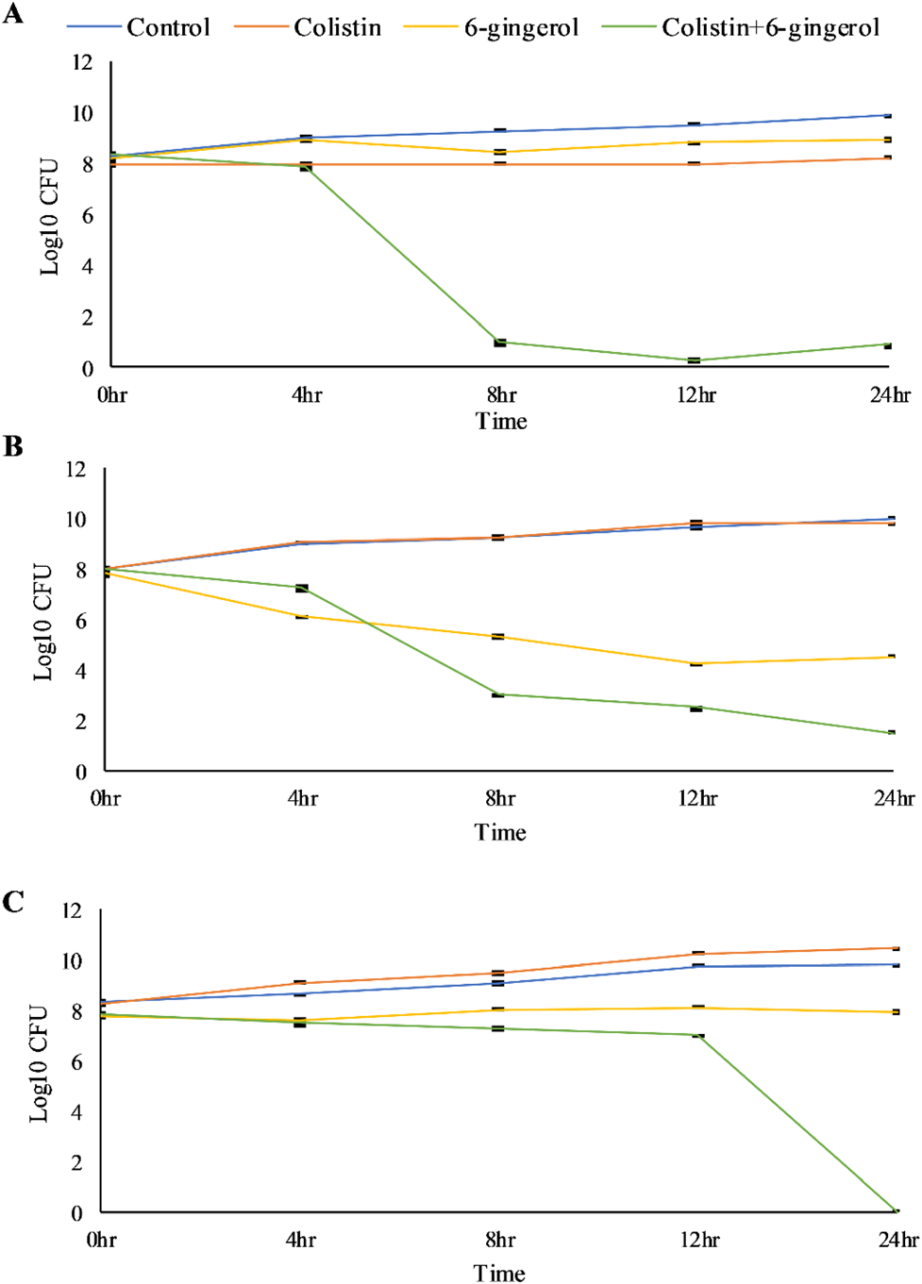
Time-kill curve of 6-gingerol combination with colistin at five different time intervals of 0, 4, 8, 12, and 24 h. The experiments were performed three times. Data are expressed as mean ± standard deviation. (A) MSK4, (B) MSK7, and (C) MSK9.

A time-kill assay was performed to validate the ability of 6-gingerol to potentiate the bactericidal effect of colistin. The result demonstrated that MSK4 and MSK7 showed a significant reduction in the CFU from 4 to 12 h with a bacteriostatic effect. In comparison, a complete reduction in CFU count after 12 h was found in MSK9, indicating the bactericidal effect (Fig. 3).

Bacteria develop antibiotic resistance and make the routine treatment critical in the infected patients.^28^ While increasing the antibiotic dose may overcome resistance but is often not recommended for toxic effects, particularly for antibiotics such as colistin. The development of resistance to colistin, the sole treatment option for infection due to MDR-KP, has made the world reach the antibiotics-era^29,30^ and prompted the use of adjuvants combined with existing treatment avenues to enhance its efficacy.^30^ Combinatorial therapy for treating these superbugs has been attempted for several reasons to replace monotherapy; primarily, its synergistic effects make the treatment more effective by preventing the emergence of new resistant strains and reducing antibiotic dose-related toxicity.^31^ In addition, it may achieve a broad spectrum of antimicrobial activity.^29,30^ Several edible natural products and food ingredients have been recently found to augment the antibacterial efficacy of nitrofurantoin and clindamycin.^32^

Herein, we screened phytocompounds of *Z. officinale* using molecular docking techniques. Based on its pharmacological properties, we identified 6-gingerol as the most promising drug candidate to target the AcrB efflux pump protein. Furthermore, we explored the antimicrobial activity of 6-gingerol against colistin-resistant MDR-KP, which could significantly reduce the MIC of colistin by exhibiting a synergistic effect. The recorded synergistic effect of the 6-gingerol and colistin against efflux-mediated colistin-resistant Gram-negative MDR-KP isolates provides the first direct, immediate evidence for the reduction of MIC of colistin through susceptibility enhancement. 6-Gingerol, the most prevailing constituent among the list of phytochemicals of ginger, has been studied to have antimicrobial activity against methicillin-resistant *Staphylococcus aureus* and *Candida albicans*.^17,33^ The 1/4 MIC of 6-gingerol alone showed bacteriostatic activity but combined with 1/4 MIC of colistin, it could considerably reduce the CFU count when compared to employing colistin alone. While various studies have investigated the biological activities of 6-gingerol and its combinatorial effects with different antibiotics, they remain unexplored. In this study, it was demonstrated that 6-gingerol enhances the antibacterial properties of colistin against MDR-KP isolates.

Currently, the enhancement in susceptibility in colistin resistance mediated by efflux pump MDR-KP isolates was observed, and it has been suggested that 6-gingerol might be inhibiting the efflux pump system responsible for bacterial resistance to colistin. Alternatively, we may hypothesize that the susceptibility enchantment could be for the enhanced influx of 6-gingerol into these bacteria due to increased permeability of the outer leaflet by colistin binding to lipopolysaccharide.^34^ However, the underlying mechanism of the natural adjuvant, 6-gingerol, which responded differentially to time-killing assay (bactericidal and bacteriostatic) to different MDR-KP strains remains unknown and warrants further investigation. Given its diverse pharmacological properties, 6-gingerol has garnered considerable attention from the scientific community. It has been increasingly incorporated as a food ingredient globally and accredited as GRAS (generally recognized as safe).^16,35^

However, this study is limited to assaying with fewer colistin-resistant MDR-KP. The *in vitro* outcome of the above combination needs further *in vivo* validation before use in clinical practice as a generalized recommendation.

## 4. Conclusion

Our results demonstrate that the combination of 6-gingerol and colistin exhibits synergistic effects against colistin-resistant MDR-KP isolates. These findings highlight the potential of 6-gingerol, a safe and natural compound, as a valuable resource for finding the links between the structure and function of antibiotic-reversing agents. Our study suggests that this compound has considerable potential for combination therapy against MDR-KP bacterial infections and can enhance the efficacy of colistin *in vitro*. These findings provide a promising alternative approach for overcoming colistin resistance. Thus, more attempts should be made to elucidate the mode of action of this compound in inciting the antibacterial activity against a larger number of MDR-KP, followed by *in vivo* toxicity and pharmacokinetics studies before recommending it as a potential adjuvant.

## Author contributions

Conceptualization: MS, ES, SD; Formal Analysis: MS, DUB, and SS; Investigation: MS, DUB, and SS; Methodology: MS, RKS; Supervision: ES; Writing original draft: MS; Writing-review, and editing: ES, and RKS.

## Conflicts of interest

The authors report that there are no competing interests to declare.

## Supplementary Material
